# A Mixed-Method Analytical Cross-Sectional Research of Person-Centered Learning Behaviors Among Adolescent and Adult Learners

**DOI:** 10.7759/cureus.61398

**Published:** 2024-05-30

**Authors:** Nirupama Bhise, Vedprakash Mishra, Sweta Pisulkar, Sharayu Nimonkar, Vikram Belkhode, Chinmayee Dahihandekar

**Affiliations:** 1 Research, Jawaharlal Nehru Medical College, Datta Meghe Institute of Higher Education and Research, Wardha, IND; 2 Physiology, Jawaharlal Nehru Medical College, Datta Meghe Institute of Higher Education and Research, Wardha, IND; 3 Prosthodontics, Sharad Pawar Dental College and Hospital, Datta Meghe Institute of Higher Education and Research, Wardha, IND

**Keywords:** person-centered learning, adult learner, adolescent learner, self-monitoring checklist, behavior

## Abstract

Background

In their academic lives, students progress from the stage of primary learning to the stage of adolescent learning and then to the stage of adult learning. At every step of learning, learners display particular learning habits, which must be mapped out to maximize learning.

Objectives

The objective of the present study is to evaluate the person-centered behaviors that influence learning among learners in adolescent and adult age groups by employing a learning behavior questionnaire that has been previously validated.

Material and methods

A cross-sectional study in which 944 participants were enrolled, including 456 adolescents from English-medium schools (aged 11 to 16 years) and 488 adults from a health professional institute (aged 18 to 23 years). The validated learning behavior questionnaire, which study participants rated on a scale of 0, 1, and 2, served as the study's quantitative component. The focus group discussion that was held for a group of adult and teenage students comprised the study's qualitative component. Using STATA-14 software (StataCorp LLC, College Station, USA), all of the responses were tallied and statistically examined.

Results

The mean scores of person-centered learning behaviors were significantly higher for learners in the adult age group than for learners in the adolescent age group. The findings of the component, which was qualitative in nature, were consistent with the findings of the learning behavior questionnaire analysis. For both adults and adolescents, the difference in mean person-centered learning scores was statistically negligible at a 5% level of significance (p=0.415 and p=0.368, respectively).

Conclusion

The study's checklist, which is self-monitoring in nature, may aid in the evaluation of learning behaviors and make it simpler for adult and adolescent learners to establish excellent learning habits.

## Introduction

A lifelong process that begins at birth and lasts until a person passes away is learning. Learning entails taking in information with all five senses, analyzing it, and assimilating the information based on willingness, belief, and personal values [1]. One of the first academics to advocate for his concept of comprehensive education to address the rapidly changing business and society was John Dewey (1938-1997) [2]. Theories of learning guide the learning process in every individual. These age-specific learning theories are crucial to understanding how a learner learns. Khalil and Elkhider studied the antecedents to learning theories based on philosophical notions, such as empiricism and rationalism, that concluded with psychological studies of functionalism and structuralism [3]. The theories of constructivism, behaviorism, and cognitivism are some examples of learning behaviors. Behaviorist theories uplift the importance of the connection between stimulus and response and describe learning as a response to natural stimuli. It further argues that answers are more likely to be repeated when they are followed by reinforcements [3]. The cognitive theories of learning place a strong emphasis on knowledge acquisition and the brain's processing of this knowledge as the fundamental elements of learning. The learner who is an active participant views information acquisition as a mental process involving coding and structure [4]. The constructivism philosophy promotes the idea that a learner acquires knowledge via their experiences and interactions. The shift of focus moves from teaching to learning along the continuum of learning theories, from a student who acts as a passive subject to a learner who acts as a proactive subject, as well as involves the passive transmission of information from the pupil to the learner's active application of knowledge [5].

Psychotherapist Carl Rogers and associates created person-centered learning behavior, sometimes referred to as person-centered counseling, person-centered psychotherapy, client-centered therapy, and Rogerian psychotherapy, starting in the 1940s and continuing until the 1980s. With the help of acceptance (unconditional positive regard), therapist congruence (genuineness), and empathic understanding, person-centered therapy aims to support a client's actualizing tendency, or "an inbuilt proclivity toward growth and fulfillment" [2].

In terms of learning behaviors, monitoring and self-regulation especially during the adolescent stage can help learners make the transition from adolescent to adult learners. Therefore, developing a self-monitoring checklist will greatly help a learner to keep track of his or her learning behaviors [6]. Teachers, facilitators, and parents can evaluate the learner's conduct using a variety of scales. For students with special educational requirements, self-monitoring scales are available; however, the demands of students generally regarding learning behaviors' self-monitoring have been disregarded, and no scale is provided for the learner himself/herself [7]. In response to the study's question - "Will an appropriate checklist for self-monitoring developed for self-assessment of behaviors meant for learning be helpful in both adult learners and adolescents?" - a self-monitoring checklist predicated on learning behaviors was developed to analyze person-centered learning behaviors, which constitutes the novelty of the study.

## Materials and methods

Study setting and design

A cross-sectional study was designed and conducted involving students from grades 6^th^ to 10^th^ at a Central Board of Secondary Education (CBSE) school as well as pupils from 1^st^ to 4^th^ year and interns at a health professional institution. The study was carried out for a period of four years from March 2016 to February 2020.

Ethical consideration

The following research received approval from the Ethics Committee of the institute in a letter dated December 30, 2015, with the reference number DMIMS (DU)/IEC/2015-16/1753. Before the research, consent was sought from the guardians/teachers of the learners in the adolescent phase and the adult learners who qualified for the inclusion criteria. The motivation for the study project was assessed for both teenage and adult learners. Participants were informed that their information would be kept private.

Sample size

N Master v2.0 (BRTC, CMC Vellore, India) was the program used to calculate the sample size. Estimated standard deviation (σ) = 32.4, required precision (d) = 0.5, and 95% confidence level (π) = 0.05 were the formulas used. The algorithm yielded a total sample size of 944 learners, of which 456 fell under the category of adolescents and 488 under that of adults. Following screening to be study participants, the 944 subjects who were included in this study responded satisfactorily and no subjects dropped out. Random sampling was done.

Data collection

A questionnaire to assess the behavior of learning was employed in the current study. The questionnaire was validated and developed [6]. The questionnaire was first designed with three learning domains in mind - cognitive, emotional, and interpersonal - and featured 17 items to be assessed on a two-point rating scale. The questionnaire was given to behavioral teaching-learning and psychology specialists for their thoughts, ideas, and suggestions. Fabricated on the ideas perceived, the questionnaire was developed and distributed to 20 7^th^-grade students, representing teenage students, and 20 4^th^-semester Bachelor of Medicine, Bachelor of Surgery (MBBS) students, representing adult students, with an equal number of female and male participants. Furthermore, the learning behavior questionnaire was validated for relevance and application on a sample that was stratified consisting of 10 pupils in every grade level of learners from adolescent and adult age groups. A conference was held and a team of four subject-matter experts from the School for Higher Education and Research Unit were assembled for validation. The specialists on the panel carefully reviewed the questionnaire, and with their approval, the scoring was assessed. A validation report for the questionnaire was provided with higher-than-average scores. Aside from the objectives that have been claimed, the behavior of the learning questionnaire included a component of qualitative aspects fabricated with learners' perceptions, in which they had to rank the assertions linked to behaviors of learning patterns in preference order. The study participants were given the verified edition of the questionnaire to assess learning behavior. The validated behavior was used to assess learning by distributing the survey to every student in grades 6^th^ to 10^th^ at a CBSE school where English was the primary language as well as to adult learners in 1^st^ to 4^th^ year and interns at a health professional institute. The responses were analyzed to identify and compare behaviors for learning assessment in both research groups. The validated questionnaire that assessed the behavior for learning patterns included 17 items regarding behaviors related to task start, continuance, and completion under the areas of “goal setting, motivation, responsibility, and self-discipline” (Table 1).

**Table 1:** Questionnaire to assess the behavior of learning

S.No	Statement	Yes	Sometimes	No
1	Do I understand my intellectual strengths and weaknesses?			
2	Am I responsible for what I learn and how well I learn?			
3	Do I ask myself questions about how well I am doing while I learn something new?			
4	Am I a good judge of how well I understand something or otherwise?			
5	Do I ask myself whether I have understood what is being taught?			
6	Do I try to find relationships between what I am learning and what I already know?			
7	Do I understand how learning differences and difficulties affect learning behavior and try to overcome them?			
8	Do I understand a topic better if audio-visual aids are used?			
9	Do I learn best when I know something about the topic?			
10	Do I try to break down large chunks of information into smaller bits for better understanding?			
11	Do I draw pictures and diagrams that help me to understand clearly while learning?			
12	Do I try to relate what I am studying to my own experiences?			
13	Do I practice applying the new principles taught in the class for remembering them?			
14	Do I translate what I have learned into my own words for better assimilation of the topic?			
15	Do I upskill others with what I have learned for better assimilation of the topic?			
16	Do I set aside a specific length of time and stick to it when I decide to study?			
17	Is learning easy in a safe and secure environment?			

Qualitative component

The qualitative component included two focus group discussions (FGD), one for learners in the adolescent phase and one for learners in the adult phase. The FGD members were chosen using computer-generated randomness. Through this method, two representatives from grades 6^th^ to 10^th^ as well as interns were chosen from the teenage age group and the adult age group.

Study population

The inclusion criteria for the current study were learners from 1^st^ year to 4^th^ year and also interns in a facility for health professionals, between 18 to 23 years, of both genders for the adult learner group. For adolescent learners, it was children from grades 6^th^ to 10^th^ of an English medium school, between 11 to 16 years, of both genders. Exclusion criteria were adolescent students in grades 6^th^ to 10^th^ in a CBSE school with English as the primary language and adult students in their 1^st^ year as interns at an institute for health professionals who were not present for three consecutive entries for the administration of the questionnaire.

Statistical analysis

For qualitative analysis, the responses of all learners from adult and teenage age groups to the questionnaire for the behavior of learning were totaled and statistically analyzed using the STATA-14 software (StataCorp LLC, College Station, USA).

For quantitative analysis of intra- and inter-group variation and significance, paired and Chi-square tests were employed.

## Results

In adult learners, values were found significant for components that were quantitative, that is, person-centered learning behaviors such as motivation, goal setting, self-discipline, and responsibility. In learners who were adolescents and adults, the rating scale of the participants for various items on the person (Pe) scale was 0, 1, and 2. The highest grade was 2. “I understand my intellectual strengths and weaknesses” (Pe1), "I am responsible for what I learn and how well I learn" (Pe2), “I ask myself questions about how well I am doing while I learn something new“ (Pe3), “I am a good judge of how well I understand something or otherwise” (Pe4), "I ask myself whether I have understood what is being taught" (Pe5), "I try to find relationships between what I am learning and what I already know" (Pe6), "I understand how learning differences and difficulties affect learning behavior and try to overcome them" (Pe7), "I understand a topic better if audio-visual aids are used" (Pe8), “I learn best when I know something about the topic” (Pe9), "I try to break down large chunks of information into smaller bits for better understanding" (Pe10), “I draw pictures and diagrams which help me to understand clearly while learning” (Pe11), "I try to relate what I am studying to my own experiences" (Pe12), "I practice applying the new principles taught in the class for remembering them" (Pe13), “I translate what I have learned into my own words for better assimilation of the topic” (Pe14), “I upskill others with what I have learned for better assimilation of the topic” (Pe15), “When I decide to study, I set aside a specific length of time and stick to it” (Pe16), and "According to me, learning is easy in a safe and secure environment" (Pe17). When compared to teenage learners, adult learners had considerably more items (p=0.05). The mean difference between person-centered learning practices in adult learners was found to be substantial when compared to teenage learners. In the adolescent learner category, person-centered learning behaviors received mean scores of 10.43 (2.98) and 10.67 (2.64) for males and females, respectively. Similarly, in the adult learners category, male students possessed a mean score of 12.18 (3.51) and female students had a score of 12.47 (3.32). The difference in mean scores for person-centered learning is statistically insignificant at a 5% level of significance in both adolescents and adults, at p=0.368 and p=0.415, respectively (Tables 2-3).

**Table 2:** Comparison of responses of adolescent and adult learners

Learning Behaviour		Observations in Adolescents	Observations in Adults
Goal setting and responsibility	Dissimilarities	Prior understanding of a subject is necessary for better learning	Prior understanding of a subject is not required for learning
		Lack of awareness of one's own advantages and disadvantages	Stated they understood their own advantages and disadvantages
		Achieving academic success mostly requires motivation, both intrinsic and extrinsic	
Motivation		Achieving academic success mostly requires motivation, both intrinsic and extrinsic	Academic success essentially requires the habit of self-motivation
		Raising the bar for academic performance might be facilitated by parents or teachers setting high goals	Establishing rigorous academic expectations from parents or educators may or may not facilitate the same
Goal setting	Similarities	Goal setting and appropriate time management do help in achieving academic success	Setting goals and practicing effective time management do contribute to academic success. The ultimate goal never changes, even while the specified goals alter from time to time
Motivation		Setting and reaching goals is aided by motivation via prizes, recognition, and gratitude	
Self-discipline		Setting goals, managing one's time, coming up with original examples related to the subject, and putting the knowledge acquired into one's own words or language all contribute to increased self-efficacy and improve comprehension and retention	

**Table 3:** Comparison of learning behaviors in adolescents and adults Coef.: Coefficient; Std. Err: Standard Errors; T: Student t-test; P value: Significant value

S.No	Learning Behavior	Adolescent (n=488) Mean (SD)	Adult (n=456) Mean (SD)	Coef. (95% Confidence Interval)	Std. Err	T	P value
Person-Centered Learning Behaviors							
1	Motivation	5.95 (2.21)	7.56 (2.54)	0.88 (0.63 – 1.12)	0.125	7.05	0.000
2	Goal setting	2.67 (1.03)	3.10 (0.98)	0.84 (0.59 – 1.09)	0.129	0.13	0.000
3	Self-discipline	7.10 (2.11)	8.64 (2.51)	1.54 (1.24 – 1.83)	0.150	10.21	0.000
4	Responsibility	5.95 (2.21)	7.56 (2.54)	0.88 (0.63 – 1.12)	0.125	7.05	0.000

## Discussion

The behavior for learning patterns that a student exhibits in the classroom frequently determines how well they succeed academically [6]. Studies on learning theories and behaviors have employed a variety of descriptors to describe learning behaviors, according to research [8]. Due to the complexity of learning behaviors and the analysis that surrounds them, there is a dearth of literature available on the topic. Thus, it is necessary to examine the concept of learning behavior. Powell and Tod conducted a systematic review earlier in 2004 in which learning behaviors were categorized as product-centered, participation-centered, and person-centered learning behaviors [1]. The close connection between 'curriculum', 'others', and 'self' as a foundation for efficient learning was highlighted by this systematic review. With the evolving trends and educational regulations, it is currently crucial to promote self-monitoring of learning behaviors to inspire students to adopt positive learning habits that will enable them to implement the necessary corrective actions to enhance their performance. As a result, a self-monitoring questionnaire was created with adult and adolescent learners in mind. There is a dearth of information regarding a cumulative assessment of the many learning behavior factors. There is research where only the impact and application of specific learning behaviors are assessed [9]. Therefore, this research was designed to include information and evaluation together with the results to complete the study's translational element.

The person-centered learning behaviors deal with the learning behaviors of the person including self-esteem, self-regard, and independent activity. These learning behaviors impact the social context of the classroom influencing collaborative learning among learners involving wholehearted involvement and full-time engagement of the learners with the help of appropriate communication. Descriptors like self-esteem, self-regard, and independent activity were used to describe the person-centered learning behaviors. The findings of the study suggest that person-in-group learning requires mutual respect among team members, responsibility towards the group activity, a conducive environment, and leadership [10]. The process of learning starts at a very early age, continues throughout the life of an individual, and is governed by learning behaviors. These learning behaviors may differ from person to person and depend on the age of the learner [11]. The statements in the study that dealt with person-centered learning behaviors are independent learning, encouragement, and self-perception/esteem/regard. The current study’s findings indicate that person-centered learning behaviors of independent learning, encouragement, and self-perception/esteem/regard have statistically significant values (p=0.00) in learners of the adult age group as compared to learners of the adolescent age group. The various theories that are proposed for person-centered learning behaviors are behaviorist theories, cognitive theories, and constructive theories. In addition to the above laws of learning, Thorndike also proposed subordinate laws of learning, which are laws of multiple responses, set attitude, analogy, associative shifting, and partial activity. These laws explain the importance of multiple ways of solving a problem, setting an attitude towards learning, learning through compare and contrast, and learning in small chunks for effective learning by a learner [12].

Albert Bandura suggested that observation, imitation, and modeling play an important role in learning. Albert Bandura stated three basic concepts that form the core of his social learning theory. He mentioned that learning can occur through observation and that the internal mental state is essential for the process of learning to occur. He also mentioned in his study that learning does not necessarily result in changed behavior [13]. Engagement, collaboration, participation, communication, motivation, independent activity, responsiveness, self-regard, self-esteem, and responsibility hereinafter are called appropriate learning behaviors. These depicted appropriate learning behaviors could be grouped into product-centered, participation-centered, and person-centered learning behaviors. Behaviors of motivation and self-discipline are referred to as basic on-task learning behaviors and are included in product-centered learning behaviors [6]. The present study also included the behaviors of goal setting and responsibility in the category of product-centered learning behaviors as these are two important learning behaviors that need to be studied separately [14]. The person-centered learning behaviors deal with the aspect of learning in groups and include behaviors like self-esteem, self-regard, and independent activity.

Studies in this particular field indicate that there is a significant association between the learners’ academic success and communication [6]. Communication in a classroom should be a two-way process and interactive. Effective communication by the teacher is a precondition to effective learning [15]. However, appropriate communication among learners also plays an important role. Restructuring the learning procedure and introducing verbal exchanges that are on-task between learners help them benefit from the more knowledgeable others in the group for enhancing their cognition and learning experience [16,17]. In the present study, the learning behavior of communication was found to have significant values for learners of the adult age group as compared to learners of the adolescent age group. The behavior of learning of responsiveness is a person-centered learning behavior dealing with the quality of speedy and positive responses of the learners. According to research, a social interactive perspective has the social context of schooling as its thrust area and is based on the ideas of social constructivism based on Piaget's and Vygotsky's beliefs [18].

Limitations of the present study are that the scope of this research is restricted to CBSE schools in which English was the primary language and a professional institute in Maharashtra, specifically in Nagpur. The research was conducted in groups of adolescent and adult students. However, research conducted according to the grade, age, person, and subject levels may be more useful in understanding behaviors of learning and transition in learners as opposed to the desired end. Meeting the requirements of the learner may unintentionally be confused with satisfying the needs or desires of the debriefer if cultural considerations are not used with awareness. The future scope of the present study is that more research can be conducted to assess the utilization of the checklist that is self-monitoring in the instillation of suitable person-centered learning behaviors in learners of adult and adolescent age groups. The creation of an Android app for learners' convenience can be done. Multicentric research ought to be conducted to assess behaviors in learning patterns in various environments and groups of age. The implication of the current study can be to set a protocol to enhance these particular learning behaviors and also to revise the curriculum to get maximum advantage for the adolescent as well as adult age groups.

## Conclusions

The study's conclusions show that gender-based differences in behavior learning in both learners of adolescent and adult age groups are not substantial. The mean scores of person-centered behaviors learning were considerably greater in students of the adult age group than in learners of the adolescent age group. The qualitative component's conclusions were consistent using the results of the study of the behavior of the learning pattern questionnaire. With the quantitative and qualitative analytical findings, a self-monitoring checklist for monitoring learning behaviors was constructed. The adult age group was more focused and self-motivated, responsible, and self-disciplined, and hence showed a higher person-centered behavior rate than adolescents. Learning behavior is a complicated structure that isn't described and seems to have arisen through the learner's triangle of interactions with the 'curriculum', 'others', and 'self', which includes parents, instructors, and classmates. Researchers have defined learning behaviors using characteristics such as involvement, cooperation, motivation, communication, engagement, and many more.

## References

[1] Powell S, Tod J (2004). A systematic review of how theories explain learning behaviour in school contexts. http://eppi.ioe.ac.uk/cms/Portals/0/PDF%20reviews%20and%20summaries/BM(CCC)_2004review.pdf?ver=2006-03-02-125203-580.

[2] Johnsen DC, Marshall TA, Finkelstein MW (2011). A model for overview of student learning: a matrix of educational outcomes versus methodologies. J Dent Educ.

[3] Khalil MK, Elkhider IA (2016). Applying learning theories and instructional design models for effective instruction. Adv Physiol Educ.

[4] Bhise N, Mishra V, Pisulkar S, Nimonkar S, Srivastava T, Belkhode V (2022). Evaluation of a product-centered learning behaviors for adolescent and adult learners using a validated learning behavior questionnaire: a mixed-method analytical cross-sectional study. Cureus.

[5] Derreumaux Y, Elder J, Suri G, Ben-Zeev A, Quimby T, Hughes BL (2023). Stereotypes disrupt probabilistic category learning. J Exp Psychol Gen.

[6] Rosenblau G, Korn CW, Pelphrey KA (2018). A computational account of optimizing social predictions reveals that adolescents are conservative learners in social contexts. J Neurosci.

[7] Bouton ME, Broomer MC (2023). Learning to stop responding. Behav Processes.

[8] Horii CV (2007). Teaching insights from adult learning theory. J Vet Med Educ.

[9] Freedman AM, Echt KV, Cooper HL, Miner KR, Parker R (2012). Better learning through instructional science: a health literacy case study in "how to teach so learners can learn". Health Promot Pract.

[10] Morisano D, Hirsh JB, Peterson JB, Pihl RO, Shore BM (2010). Setting, elaborating, and reflecting on personal goals improves academic performance. J Appl Psychol.

[11] Asci H, Kulac E, Sezik M, Cankara FN, Cicek E (2016). The effect of learning styles and study behavior on success of preclinical students in pharmacology. Indian J Pharmacol.

[12] Elliot AJ, Thrash TM, Murayama K (2011). A longitudinal analysis of self-regulation and well-being: avoidance personal goals, avoidance coping, stress generation, and subjective well-being. J Pers.

[13] Bandura A (2004). Health promotion by social cognitive means. Health Educ Behav.

[14] Murayama K, Elliot AJ (2011). Achievement motivation and memory: achievement goals differentially influence immediate and delayed remember-know recognition memory. Pers Soc Psychol Bull.

[15] Carter AG, Creedy DK, Sidebotham M (2017). Critical thinking skills in midwifery practice: development of a self-assessment tool for students. Midwifery.

[16] Descals-Tomás A, Rocabert-Beut E, Abellán-Roselló L, Gómez-Artiga A, Doménech-Betoret F (2021). Influence of teacher and family support on university student motivation and engagement. Int J Environ Res Public Health.

[17] Wills HP, Mason BA (2014). Implementation of a self-monitoring application to improve on-task behavior: a high school pilot study. J Behav Educ.

[18] Forman EA, Kraker MJ (1985). The social origins of logic: the contributions of Piaget and Vygotsky. New Dir Child Dev.

